# Spatial distribution and source apportionment of DTPA-extractable metals in soils surrounding the largest Serbian steel production plant

**DOI:** 10.1016/j.heliyon.2023.e16307

**Published:** 2023-05-15

**Authors:** Snežana Dragović, Ivana Smičiklas, Mihajlo Jović, Aleksandar Čupić, Ranko Dragović, Boško Gajić, Antonije Onjia

**Affiliations:** a "VINČA" Institute of Nuclear Sciences – National Institute of the Republic of Serbia, University of Belgrade, Mike Petrovića Alasa 12-14, 11351 Belgrade, Serbia; b Department of Geography, Faculty of Sciences and Mathematics, University of Niš, Višegradska 33, 18000 Niš, Serbia; c Faculty of Agriculture, University of Belgrade, Nemanjina 6, 11080 Belgrade, Serbia; d Faculty of Technology and Metallurgy, University of Belgrade, 11120 Belgrade, Serbia

**Keywords:** Potentially toxic elements, Steel production, Bioavailability, Soil pollution, Multivariate analysis, Gis

## Abstract

Despite presenting a practical approach for the characterization of the environmental risk of potentially toxic elements (PTEs) derived from steel production facilities, the analysis of the spatial distribution of bioavailable PTEs concentrations in the soil is frequently overlooked in the management of polluted sites. In this study, the diethylenetriaminepentaacetic acid (DTPA)-extractable forms of PTEs were investigated in soils surrounding the largest Serbian steel production plant. The correlation and geostatistical analysis indicated their pronounced variability suggesting the anthropogenic origin of most investigated elements, apparently from the steel production facility. The detailed visualization of variables and observations derived by self-organizing maps (SOMs) revealed the homologies in PTEs’ distribution patterns, implying the common origin of some elements. These observations were confirmed by principal component analysis (PCA) and positive matrix factorization (PMF). The аpplied approach supports a comprehensive assessment of contaminated sites' ecological and health risks and provides a basis for soil remediation.

## 1 Introduction

Iron and steel production is one of the essential mineral processing industries with a significant contribution to global economic development [1]. However, the activities associated with this industry pose considerable environmental risks. The acidic gaseous pollutants, incomplete combustion pollutants, particulate matter, and potentially toxic elements (PTEs) are generated from exhaust gas during iron and steel production [2]. In addition, the soils surrounding steelmaking facilities can be contaminated by major ore metals, metals contained in ore minerals as minor inclusions, and toxic waste [[3], [4], [5], [6]]. Metal processing and mining contribute to almost half of the total release of contaminants by the European industrial sector, with PTEs being the primary pollutants in European soils and groundwater [7]. Therefore, the balance between the economic input of the steelmaking industry and its environmental impact should determine its contribution to sustainable development.

The PTEs are chemical elements of great environmental concern due to their persistence in both abiotic and biotic compartments, and a series of adverse effects on human health and the environment triggered at certain concentration levels. In the steel production process, the integrated coal-fired boiler and smelting furnace, where PTEs in fuels and ores are usually converted into metal oxides or chlorides [8], are the most important pollution sources. The diffusion distance of PTE emissions from steel industrial facilities depends on particle size distribution, dominant wind direction, stack height, and proportion of mercury and arsenic in the gaseous phase [9]. In addition, the iron and steelmaking processes produce waste products, such as slag being generally considered one of the main sources of contamination by PTEs in soil, water, and sediments near the slag deposition sites [10,11] and off-gas that is subjected to gas treatment [12]. However, the gas cleaning systems work under limited efficiencies, releasing some volatile trace metals to the environment through atmospheric processes, particle emissions, and subsequent deposition on the soil surface. In soils, these elements are found in different fractions, in mobile or immobilized forms, i.e., as exchangeable, carbonate-bound, Fe and Mn oxide-bound, organic-bound, and residual forms [13,14]. Soil properties, such as acidity, redox potential, and dissolved organic matter, influence metal dynamics in soils [15]. The speciation of PTEs in soil plays a key role in their distribution, persistence in the environment, and bioavailability. Since there is no official definition of the term PTE, it should be noted that toxicity depends on several factors, including the type of element, its chemical form and dose, and the specifics of the exposed organism. While metals with no biological role are toxic even in very low concentrations, some (e.g., Fe, Mn, Zn, Cu, Co, Mo) are essential for living organisms, particularly for chemical and biological activities [16]. Still, after exceeding a specific threshold limit, they can also cause either short-term or long-term toxic impacts [17].

The current approach to evaluating potential environmental and human health risks based on the total concentrations of PTEs in soil was found to be inadequate and insufficient [[18], [19], [20], [21]]. However, based on bioavailability, it is possible to predict the transport, fate, and potential environmental impact of PTEs [22]. Therefore, in this study, the impact of the steel production facility was assessed by analysis of bioavailable PTE concentrations in soils of the surrounding area. The specific objectives of the study were (i) to determine the spatial distribution of DTPA-extractable PTEs in the topsoil surrounding the steel production plant, (ii) to investigate their mutual relationships and relationships with basic soil characteristics and geographical factors, and (ii) to identify the sources’ contribution of soil PTEs by principal component analysis (PCA), self-organizing maps (SOM), and positive matrix factorization (PMF). The study outcomes will contribute to developing appropriate measures to mitigate pollution by PTEs by including their bioavailable concentrations in the risk assessment protocols.

## 2 Materials and methods

### 2.1 Study area and sample collection

The industrial zone of the integrated steel plant, producing steel, hot-rolled, and cold-rolled products, is located in central Serbia, about 7 km southeast of the town Smederevo and about 50 km of Belgrade, the Serbian capital. Geologically, the terrains of the Smederevo area belong to Vardar Zone and Serbian-Macedonian Mass. The study area is presented with white quartz sand, sandy clays with intercalations, and clay sands with diverse fauna and coal occurrences. In the wider Smederevo area, the sandy-gravel series is located between Pontian deposits and Pleistocene sediments. The Quaternary formations in the facies of Pleistocene are widely distributed in most parts of the area. The Holocene formations are presented by fluvial and slope sequences [23].

The climate is moderate continental, characterized by hot summers with high precipitation in June and November and cold, dry winters. According to the Köppen classification, the climate type is Cfwax [24]. From 1949 to 2021 the mean annual values of temperature, precipitation, and humidity were 11.5 °C, 649 mm, and 72%, respectively [25]. The main soil types of the study area are Eutric Cambisols (brown soils), Vertisols (clay-rich soils with shrink-swell properties), Colluvic Regosols (soils formed on slope deposits), Phaeozems (humid steppe soils), and Fluvisols (soils formed from river deposits) [26].

**Fig. 1:**
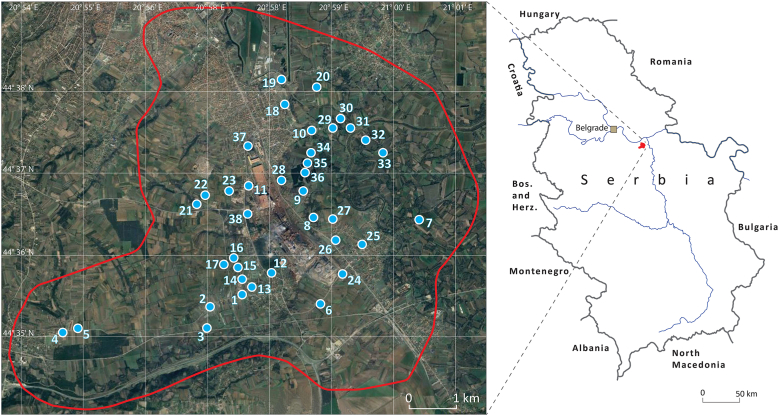
Simplified maps showing the position of the study area within Serbia and the location of sampling sites.

Soil samples were collected in October 2019 from 38 sampling sites (0–10 cm depth) applying the ‘systematic random sampling’ procedure [27] in the steel production facility's surroundings potentially affected by industrial activities (Fig. 1), with the majority of sampling sites in the most densely populated area. The geographic coordinates of the sampling sites were obtained via the Global Positioning System (Table S1). The samples were taken in open/unoccupied land away from roads and power cables to avoid contamination.

About 1.5 kg of each sample was collected using clean disposable gloves, a stainless steel spade, and a plastic scoop. To avoid cross-contamination, the equipment was first brushed to eliminate residues from the previous sample and then flushed with soil from the new sampling site. To ensure the representativeness of the specimen at each site, three subsamples were collected at distances of about 10 m between each other.

### 2.2 Soil physicochemical analyses

Before physicochemical analyses, soil samples were dried at room temperature, ground, and passed through a 2 mm stainless steel sieve. Each sample was then homogenized and ground in a mortar.

The traditional sieve-pipette method was used for particle size analysis [28].

Soil organic matter (OM) content was determined by the loss-on-ignition (LOI) method, which involves the destruction of soil OM by heating [29]. First, the portions of 10 g of soil samples (oven-dried at 105 °C till constant weight) were precisely measured in ceramic crucibles of known weight and heated in the electrical furnace at 400 °C for 17 h. The temperatures below 440 °C are favorable to prevent the decomposition of inorganic carbonates if present in the soil. After cooling in a desiccator, the crucibles were weighed again, and the SOM (%) was calculated.

The pH of soil samples was measured in water at a 1:1 soil-to-water ratio (w/v) according to the US EPA 9045D method [30]. The 10 g portion of each soil sample and 10 mL of deionized (DI) water were stirred continuously in a beaker on the magnetic stir plate for 5 min and then left to settle down the suspended particles (1 h). The pH was measured by immersing the pH electrode just below the suspension and allowing the pH reading to stabilize. The InoLab WTW pH meter, equipped with the WTW SenTix® 81 glass electrode, was used and calibrated with buffer solutions (pH 4, pH 7, and pH 10). Calibration was verified after every ten samples analyzed.

The bioavailable concentrations of Cd, Co, Cu, Fe, Mn, Ni, Pb, and Zn were determined following the soil extraction with DTPA [31,32]. The extracting solution was prepared from 0.005 mol L^−1^ DTPA, 0.1 mol L^−1^ triethanolamine (TEA), and 0.01 mol L^−1^ CaCl_2_, adjusting the final pH of the solution to 7.3 with 6 mol L^−1^ HCl. Each soil sample (10 g) was extracted with 20 mL of the buffered DTPA solution during 2 h of shaking on a rotary shaker (15 rpm) at room temperature (25 ± 1 °C). Subsequently, the liquid phase was separated from the solid by membrane filtration (PTFE syringe filters, pore size 0.45 μm), and the metal concentrations in the extracts were measured within 24 h by Inductively Coupled Plasma Optical Emission Spectroscopy, ICP-OES (PerkinElmer Avio 200). The multi-element calibration standards were used to prepare the matrix-matched standards with concentrations in the range 0.01–1.0 mg L^−1^ of Cd; 0.01–5 mg L^−1^ of Co, and Ni; 0.1–10.0 mg L^−1^ of Cu, Zn, and Pb; 0.5–50.0 mg L^−1^ of Mn; 1.0–100.0 mg L^−1^ of Fe. Linear calibrations were obtained for all tested elements, with calibration coefficients >0.999. The quality of the metal concentrations analyses was assured by including duplicate extractions of soil samples, three replicate measurements of blank and sample solution, and calibration checks after every ten samples analysis. Concentrations of elements measured in the extracts are presented in mg kg^−1^ of the soil on a dry matter basis.

### 2.3 Geostatistical analysis

Geostatistics was employed to gain insight into the overall spatial pattern of analyzed variables over the study area. Geostatistics is based on the theory of a regionalized variable [33], which shows spatial autocorrelation where observations close to each other are more alike than those far apart. As an interpolation method, kriging predicts values in areas where data are unavailable based on the spatial patterns of the existing data by the semivariogram model. A semivariogram indicates the spatial autocorrelation and variation of the soil samples, which are defined as half of the averaged squared difference between the paired data values separated by a distance interval as in Eq. (1) [33]:

(1) $$\gamma \left(h\right)=\frac{1}{2N\left(h\right)}\sum_{i=1}^{N\left(h\right)}\left\{Z\left(X_{i}\right)-Z\left(X_{i}+h\right)\right\}$$

where γ(h) represents the semivariogram value, *N*(*h*) denotes the number of pairs of sample points, and *Z* (*Xi*) and *Z(Xi + h)* are the numerical values measured at locations *Xi* and *(Xi + h)*, respectively, and is the numerical value at a distance *h* from *Xi*. Four types of semivariogram models (circular, spherical, exponential, and Gaussian) were tested. Cross-validation was used to compare the measured and predicted values for each variable.

### 2.4 SOM

The SOM is an unsupervised learning artificial neural network model which classifies the input pattern set by finding the optimal reference vector set. The first step in SOM classification is an ordered dimensionality-reducing mapping of input variables while preserving the original topology of the input data [34]. The neurons located near each other in the SOM had similar associated input samples. During the iterative learning procedure, the SOMs were trained for all possible combinations from 2 to 20 neuron nodes for the X and Y axes. The quality of these alternative topologies was evaluated using the quantization error (QE), which evaluates the resolution of the map, and the topographic error (TE), which indicates the accuracy of the topology preservation of the map [35,36]. The learning rate (α) varied linearly from 0.05 to 0.01, and the neighborhood function was Gaussian. Finally, the input sample data were presented to each SOM 500 times, and the selected distance measure was the Euclidean distance. Once the optimal dimensions of the SOM were determined based on the QE and TE, Ward's linkage method was applied to the SOM to cluster the unit neurons and, consequently, the soil samples. The relevant patterns are graphically displayed using component planes. The hierarchical classification was used to reduce the number of classes to a small number of superclasses. This double classification enables an analysis of the data set at two levels, at first enabling precise characterization of the data and at the second level where general features emerge.

### 2.5 Correlation analysis, PCA, and PMF

The Pearson correlation matrix was used to determine the type of correlations among analyzed variables.

A PCA pattern recognition method was used to reduce the dimensionality of input data and transform interdependent variables into independent principal components (PCs) as shown in Eq. (2):

(2) $${PC}_{k}=a_{1k}X_{1j}+a_{2k}X_{2j}+a_{3k}X_{3j}+\ldots +a_{nk}X_{nj}$$

whilst the sum of the coefficients a_ik_, called loadings, is set to unity [37]. The loading of a single variable indicates how much that variable participates in defining the PC (the squares of the loadings indicate their percentage in the PC). This is done in such a way as to minimize the loss of information carried by variables. The PCs comprise loadings normalized eigenvectors between +1 and −1, considered as the subspace coordinates of data and scores, which are the sample values projected on the subspace using the eigenvectors. The technique has three effects: it orthogonalizes the components of the input vectors (so that they are uncorrelated with each other), it orders the resulting orthogonal components (principal components) so that those with the largest variation come first, and it eliminates those components which contribute least to the variation in the data set.

The source apportionment was performed using the PMF model. Briefly, the PMF model is based on the decomposition of matrix X (*m* × *n*) of measured data into a factor contribution matrix G (*n* × *p*) and factor profile matrix F (*p* × *m*) with a residual error matrix E, where *n* is a number of samples, *m* is the pollutant component, and *p* is the number of pollution sources. The PMF solves the chemical mass balance between source profiles and variables measured values as in Eq. (3):

(3) $$x_{ij}=\sum_{k=1}^{p}g_{ik}f_{kj}+e_{ij}$$

where *x*_*ij*_ is the *j*th measured value of the variable in the *i*th sample, *g*_*ik*_ is the mass of the *k*th source to the *i*th sample, *f*_*kj*_ is the *j*th species profile in source *k*, and *e*_*ij*_ is the matrix of the residual associated with the *j*th species measured value in the *i*th sample. The profile and contribution of each source are derived by minimizing the object function Q as shown in Eq. (4) [38]:

(4) $$Q=\sum_{i=1}^{n}\sum_{j=1}^{m}.{\left[\frac{x_{ij}-\sum_{k=1}^{p}g_{ik}f_{kj}}{u_{ij}}\right]}^{2}$$

where Q is the sum of the squares of the difference between the original dataset and the PMF output; u_ij_ represents the measurement uncertainty.

### 2.6 Statistical analysis

Basic descriptive statistics of variables, correlation analysis, and PCA were performed using SPSS 10.0 software. The variables were geostatistically mapped using the Surfer program from Golden software. The training and visualization of the SOM were performed using the functionalities implemented within the R Package Kohonen. The distribution of wind direction frequencies in the study area in 2019 was presented using the R Package Openair. The softver EPA PMF 5.0 was used for PMF.

## 3 Results

### 3.1 Descriptive statistics, spatial distribution, and correlation analysis of basic soil characteristics and DTPA-extractable PTEs in the steel production facility's surroundings

Descriptive statistics of basic soil characteristics is summarized in Table 1 (the complete characterization of soil samples is given in Tables S2 and S3). The OM content ranged from 0.69% to 4.44%. Soil pH ranged from medium acidity (5.94) to medium alkalinity (8.04). Based on USDA's (1998) classification [39], the soils were classified as silt loam (36.8%), silty clay loam (30.0%), loam (23.7%), and clay loam (10.5%).

**Table 1:** Descriptive statistics of basic soil characteristics.

Parameter	Sand (%)	Clay (%)	Silt (%)	OM (%)	pH
Mean	20.8	24.0	55.3	2.00	7.40
Standard deviation	13.6	4.58	13.2	0.77	0.51
Variance	184.7	20.94	173.2	0.60	0.26
Coefficient of variation (%)	65.4	19.1	23.8	38.7	6.94
Minimum	4.1	14	26.2	0.69	5.94
Maximum	48.3	32.5	74.6	4.44	8.04
Median	18.7	23.3	58.0	1.96	7.52
Range	44.2	18.5	48.4	3.75	2.1
Skewness	0.44	−0.11	−0.50	0.79	−1.17
Kurtosis	−1.04	−0.85	−0.81	1.43	1.16

The descriptive statistics summary of soil bioavailable PTE concentrations in the steel production facility's surroundings is presented in Table 2. The mean bioavailable PTE concentrations followed the order: Mn > Fe > Cu > Zn > Ni > Pb > Co > Cd. The skewness values higher than 1 for all analyzed elements show that these elements positively skew towards lower concentrations. Kurtosis values of all elements above 0 indicate that the distribution of elements is steeper than normal.

**Table 2:** Descriptive statistics of soil bioavailable PTE concentrations (mg kg^−1^) in the steel production facility's surroundings.

Parameter	Cd	Co	Cu	Fe	Mn	Ni	Pb	Zn
Mean	0.07	0.12	3.75	28.9	37.2	2.88	2.85	3.25
Standard deviation	0.060	0.086	4.606	23.68	18.034	2.975	2.823	2.603
Variance	0.004	0.007	21.22	560.8	325.24	8.853	7.968	6.775
Coefficient of variation (%)	85.7	71.7	122	81.9	48.5	103	99	80
Minimum	0.01	0.01	0.60	8.29	14.19	0.47	0.81	0.62
Maximum	0.39	0.47	28.4	97.7	89.1	15.2	17.7	14.0
Median	0.06	0.10	2.31	19.5	33.0	1.83	2.13	2.48
Range	0.38	0.47	27.8	89.4	74.9	14.7	16.9	13.4
Skewness	3.98	2.08	4.47	1.68	1.32	2.60	4.27	2.39
Kurtosis	20.5	7.04	23.2	2.25	1.81	7.69	21.5	7.28

**Fig. 2:**
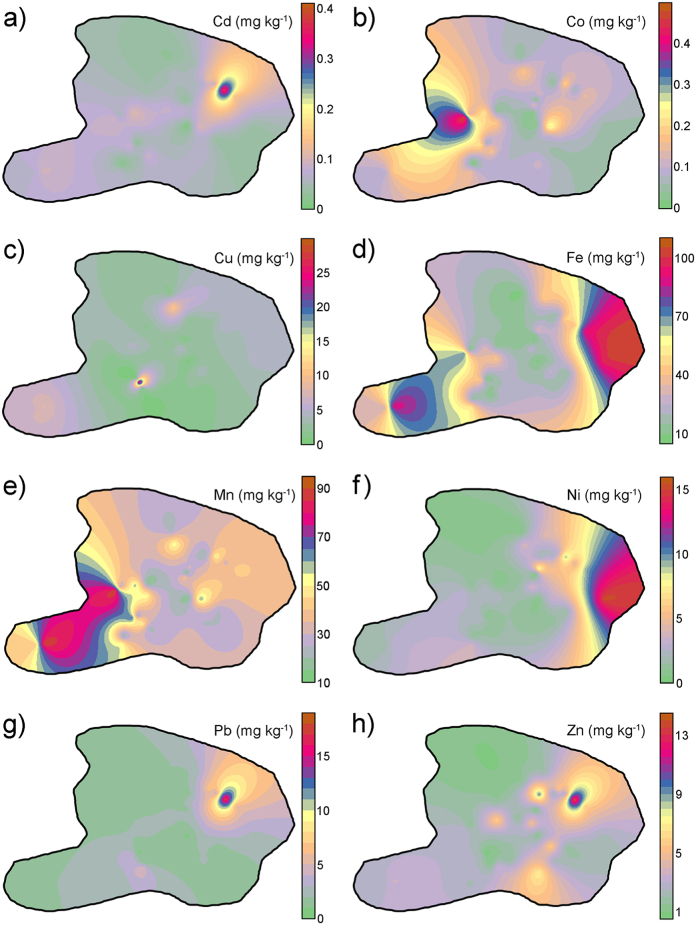
Maps of bioavailable PTE concentrations of a) Cd, b) Co, c) Cu, d) Fe, e) Mn, f) Ni, g) Pb, and h) Zn in the steel production facility's surroundings obtained by ordinary kriging.

An ordinary kriging interpretation was used to map the spatial distribution of the PTEs in the soil of the study area and identify pollution hotspots. The spatial variations of the PTE concentrations in the soils from the steel production facility area are shown in Fig. 2. The various colors indicate the DTPA-extractable PTE concentrations. The red color stands for high concentration, whereas the green color stands for low concentration. As evident from the geospatial maps (Fig. 2), the element concentrations in the investigated area exhibited significant metal-specific and site-specific variability, suggesting a strong influence of anthropogenic sources on the metal concentrations in the area [40]. The Cd, Pb, and Zn distributions (Fig. 2, a, g, and h) have similar geographical patterns, with hotspots in the eastern and northeastern parts of the investigated area. Cobalt and Mn (Fig. 2, b, and e) show the highest concentrations in the western part of the area. Nickel (Fig. 2, f) was primarily distributed in the eastern part of the investigated area. Iron (Fig. 2, d) was widely distributed in the area, with hotspots in its east and southwest parts.

As soil properties such as soil texture, OM content, and pH may impact the distribution and bioavailability of PTEs in soil [21,41,42], their relationship with PTEs is analyzed in this study. The correlation matrix (Table 3) was used to estimate the strength of the relationships among analyte pairs in the soil samples. It revealed a significant positive correlation between Cd, Pb, and Zn. Cobalt and Mn showed a positive mutual correlation, while Fe was significantly positively correlated with Mn and Ni. Cobalt showed a significant positive correlation with silt, and Ni was positively correlated with sand and negatively correlated with silt.

**Table 3:** The correlation matrix for DTPA-extractable PTEs and soil physicochemical characteristics.

	Sand	Clay	Silt	OM	pH	Cd	Co	Cu	Fe	Mn	Ni	Pb
Clay	−0.261											
Silt	−0.942^a^	−0.078										
OM	−0.276	0.042	0.270									
pH	0.168	−0.358^b^	−0.049	−0.504^a^								
Cd	−0.077	0.204	0.008	0.296	−0.145							
Co	−0.428^a^	0.016	0.437^a^	0.621^a^	−0.688^a^	0.127						
Cu	−0.227	0.346^b^	0.114	0.136	−0.117	0.067	0.082					
Fe	0.087	0.303	−0.195	0.393^b^	−0.814^a^	0.282	0.385^b^	0.076				
Mn	−0.448^a^	0.231	0.382^b^	0.525^a^	−0.870^a^	0.201	0.868^a^	0.208	0.599^a^			
Ni	0.440^a^	0.232	−0.535^a^	0.239	−0.456^a^	0.179	0.099	0.050	0.723^a^	0.213		
Pb	0.212	0.192	−0.285	0.178	−0.004	0.893^a^	−0.096	−0.002	0.206	−0.024	0.261	
Zn	0.209	0.129	−0.261	0.083	−0.004	0.765^a^	−0.072	−0.035	0.210	−0.019	0.161	0.703^a^

a Correlation is significant at the 0.01 level.

b Correlation is significant at the 0.05 level.

### 3.2 Classification by SOM

The visualization and classification were obtained by the SOM of six rows and six columns with a hexagonal topology. A bubble neighborhood function was trained on 11 variables (PTEs and basic soil properties) and 38 observations (soil samples). The SOM simultaneously minimized the quantization and topographic errors to 0.95 and 0.05, respectively. After multiple rounds of iterative training, the weight matrix of each soil PTE is obtained.

**Fig. 3:**
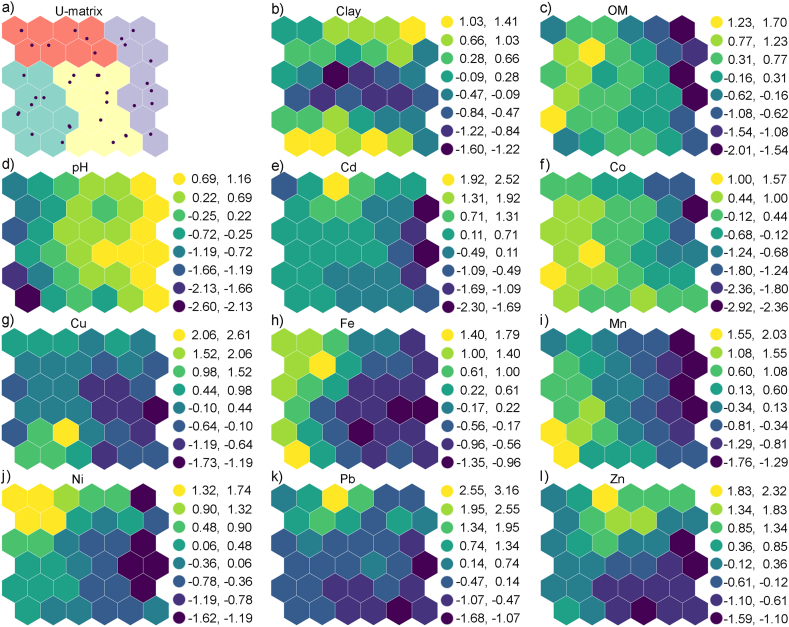
SOM visualization of basic soil characteristics and bioavailable PTEs in soils of the steel production facility's surroundings: a) U-matrix and component planes for b) clay, c) OM, d) pH, e) Cd, f) Co, g) Cu, h) Fe, i) Mn, j) Ni, k) Pb, and l) Zn.

**Fig. 4:**
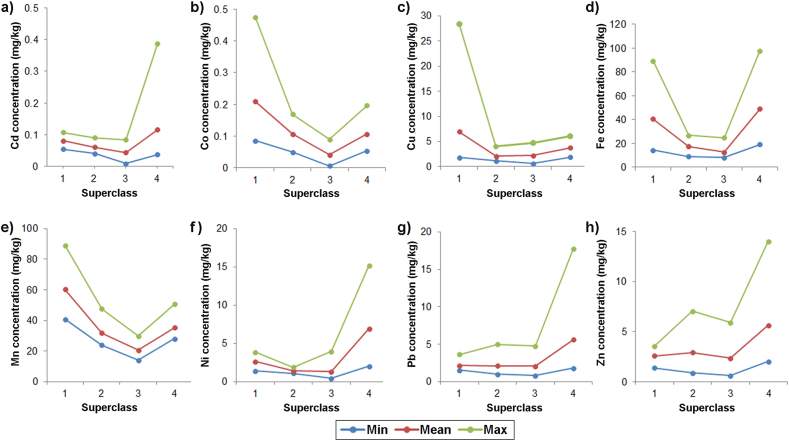
Minimum, mean, and maximum values of DTPA extractable PTE concentrations of a) Cd, b) Co, c) Cu, d) Fe, e) Mn, f) Ni, g) Pb, and h) Zn in SOM superclasses.

The SOM results are presented in Fig. 3, which shows the unified distance matrix (U-matrix, Fig. 3, a), followed by component planes, one for each variable (Fig. 3, b-l). The U-matrix represents the Euclidean distance between the weight vectors of neighboring neurons on the map in the color scale. In component planes, the weight vector values of each neuron are represented by different colors, with yellow and blue representing high and low parameter values, respectively. The neuron color similarity represents significant correlation between parameters. Cobalt, Fe, Mn, and Ni have a similar color pattern (Fig. 3, f, h, i, and j), i.e., high-value distribution in the left part of the map. Cadmium, Pb, and Zn (Fig. 3, e, k, and l) have a high-value distribution in the middle upper part of the grid, indicating the positive associations between these elements. According to the U-matrix (Fig. 3, a), the sampling points with basic soil characteristics and PTEs concentrations are divided into four superclasses. The contents of elements among superclasses are further analyzed as shown in Fig. 4.

### 3.3 Source identification by PCA

The PCA was applied to further identify the source of analyzed PTEs in the soils of the study area. The multivariate data set consists of 11 variables (3 independent soil basic characteristics and 8 PTEs) measured over 38 soil samples (observations), yielding a 38 × 11 (rows by columns) primary data matrix. Since the variables' different ranges and units and their deviation from a normal distribution, the data was log-transformed before the analysis. The number of significant PCs was selected based on the Kaiser criterion [43], retaining PCs with eigenvalues that exceed one. The scree plot test consists of plotting the eigenvalues against the number of the extracted components and finding the points where the smooth decrease of eigenvalues appears to level off to the right of the plot, eliminating components that contribute to factorial scree only. The scree plot for the given data set (Fig. S1) showed that three components accounting for most of the total variance complied with the Kaiser criterion. To gain a better insight into the data's hidden regularities and ensure that the resulting factors were uncorrelated, the PCA extracted correlation matrix was subjected to varimax (variance maximizing) orthogonal rotation. The significant factors obtained after varimax rotation and loadings indicating how each analyte is related to these factors are presented in Table 4. Factor 1, explaining 32.0% of the total variance, showed high factor loadings of OM, pH, Co, Fe, and Mn. Factor 2 (24.0% of the total variance) showed high factor loadings of Cd, Ni, Pb, and Zn. Factor 3 (18.9% of the total variance) showed high factor loadings of clay and Cu.

**Table 4:** Varimax rotated factor loadings and communalities of soil physicochemical characteristics and DTPA-extractable PTE concentrations in soils in the steel production facility's surroundings

Variable	Factor 1	Factor 2	Factor 3
Clay	-0.024	-0.001	**0.842**
OM	**0.776**	0.314	-0.055
pH	**-0.762**	0.003	-0.497
Cd	0.402	**0.768**	0.149
Co	**0.915**	0.009	-0.037
Cu	0.138	0.230	**0.668**
Fe	**0.559**	0.373	0.555
Mn	**0.908**	0.088	0.276
Ni	0.431	**0.498**	0.497
Pb	0.006	**0.881**	0.103
Zn	0.016	**0.851**	0.109
Variance	3.52	2.64	2.08
% Var	32.0	24.0	18.9

The highest absolute value of loadings for each variable is shown in bold

### 3.4 Source apportionment by PMF model

For further analysis of the type of source apportionment and the contribution rate of PTEs in soil samples around the steel production facility, the PMF model was applied. The PMF model was constructed using basic soil characteristics and PTEs concentrations. The factor number of 2–6 in the base PMF model was consecutively selected until the Q value was at a minimum. The number of runs was set to 20, and the start seed number was chosen randomly. The smallest and most stable Q value was obtained when the number of source factors was set to 4. Thus, most of the values in the residual matrix varied within ±5. The correlation coefficients between the measured and estimated concentrations ranged from 0.68 to 0.99, indicating that the PMF model apportioned analyzed PTEs with a reasonable number of factors to explain the raw data fully.

**Fig. 5:**
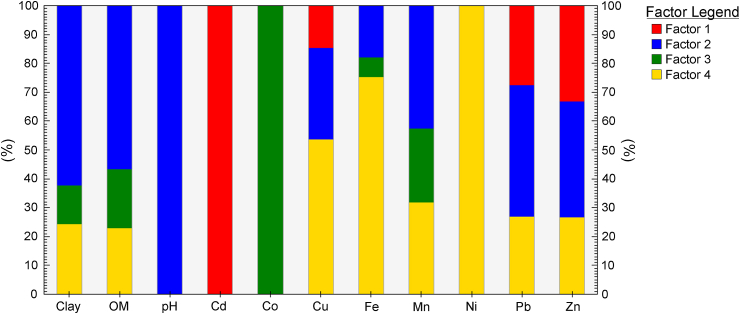
PMF factor fingerprints of bioavailable metal concentrations and basic physicochemical characteristics of soils.

The PMF factor fingerprints showed that all basic soil characteristics and PTEs (Fig. 5), except pH, Cd, Co, and Ni, were influenced by three factors. Factor 1 contributed to Cd (100%), Cu, Pb, and Zn. Factor 2 mainly contributed to pH (100%), clay, and OM. Factor 3 contributed to Co with 100% and to less extent to Mn and OM. Factor 4 was the dominant contributor of Ni (100%), Cu, and Fe.

## 4 Discussion

**Fig. 6:**
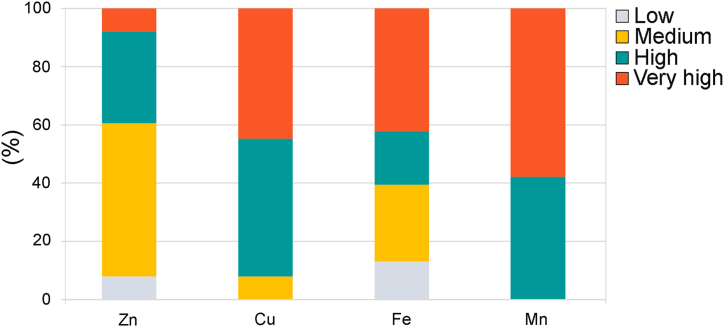
Percentage distribution of the essential micronutrients' levels in soil samples (n = 38) according to [46].

The DTPA extraction method is applicable for assessing micronutrients (Fe, Mn, Zn, and Cu) availability to plants in neutral and alkaline soils [31]. The buffered DTPA extraction solution forms strong heavy metal-DTPA complexes isolating their water-soluble, exchangeable amounts and specifically adsorbed forms that are available to plants [44]. Furthermore, the 0.005 M DTPA solution was recommended as an extractant for determining the geochemical reactivity of PTEs in contaminated soils [45]. It is also a reliable method for determining deficiencies in micronutrients in soil which influence crop growth and reduce their quality and potential yield. In this respect, Barrett et al. (2017) provided general recommendations for DTPA-extracted concentrations of micronutrients needed by plants and, accordingly, the soil micronutrients’ status classification as very low, low, medium, high, and very high [46]. Based on that classification, the mean bioavailable concentrations (Table 2) levels of Cu, Fe, and Mn in soils analyzed in this study are very high, and those of Zn are high. The distribution of micronutrients levels (Fig. 6) highlights a small number of samples with low levels of Zn (7.8%) and Fe (13.1%) in contrast to a high proportion of soils with high and very high levels of Fe (18.4% and 42.1%) and particularly Mn (42.1 and 57.9%) and Cu (47.4% and 44.7%).

The CV values of all analyzed PTEs reflect their high variability in the soils of the investigated area and suggest that the investigated area could be significantly affected by anthropogenic activities [[47], [48], [49]]. The concentrations of Co, Cu, and Zn obtained in this study are consistent with those for topsoils surrounding a steel industrial area in Iran, reported to be up to 2.4, 9.2, and 10 mg kg^−1^, respectively [50]. The DTPA-extractable concentrations of Cd, Cu, Pb, and Zn in analyzed soils are still lower than those in soils of the area in the North China Plain receiving the effluents from smelter plants on a long-term basis, reported to be up to 1.58, 210, 12.3, and 372, respectively [51]. Even when production activities are deceased, the bioavailable concentrations of PTEs in soils surrounding steelmaking facilities could remain high. Thus, bioavailable concentrations of Cd and Pb above values considered safe by international standards have been found in soils contaminated by slag from an abandoned steel plant in Havana, Cuba [6].

**Fig. 7:**
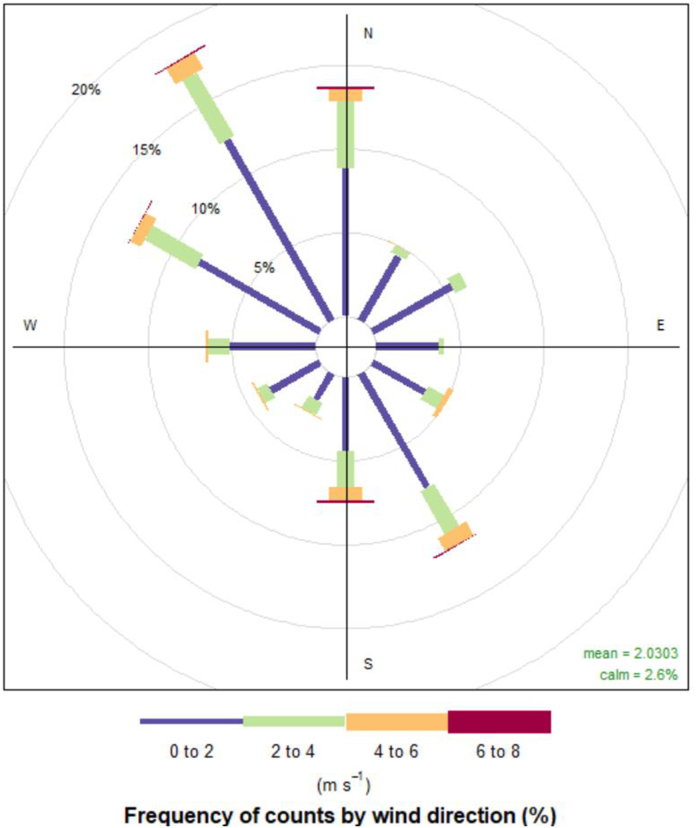
The distribution of wind direction frequencies in the study area (annual average for 2019).

The maps derived by ordinary kriging indicate the PTE spatial distribution in the investigated area. To further explain the PTEs’ spatial patterns in soils surrounding the steel production facility, the influence of prevailing winds on their distribution was investigated. The wind is the dominant meteorological parameter for the air distribution of pollutants emitted from industrial chimneys. The distribution of wind direction frequencies (the annual average for 2019) calculated from data obtained from the Smederevska Palanka meteorological station (44°22′ N, 20°57′ E) is shown in Fig. 7. The direction distribution in the wind rose indicated the prevalence of winds from the second (E-S) and fourth (W–N) quadrants. Thus, the predominant winds are WNW and ESE, blowing from the northwest and southeast. The distribution of Fe and Ni could result from prevailing winds, as the highest concentration is in the eastern part of the investigated area. A similar situation occurred with Cd, Pb, and Zn. The results obtained in this study are in accordance with the findings of Odabasi et al. (2010) [52], who reported the highest concentrations of PTEs in the soil around iron-steel plants at points where prevailing winds transport industry emissions. Zhou et al. (2019) reported the highest concentrations of Pb and Zn in soils in the prevailing downwind direction from the iron and steel plant in North China Plain [53]. The spatial distribution analysis of the PTEs in urban soils from an industrial district in Northeast China revealed their pollution by Cu, Pb, and Zn with contamination hotspots in the steel industrial district [54].

The correlation analysis results were consistent with the spatial distribution characteristics of the analyzed elements. The elements showing similar spatial patterns in the investigated area, such as Cd, Pb, and Zn (Fig. 2, a, g, and h), were highly correlated (Table 3). Iron and Mn, highly correlated according to Table 3, show similar spatial distribution (Fig. 2d and e). The similar distribution patterns of Co and Mn (Fig. 2, b and e) are also confirmed by correlation analysis. It is well-recognized that many physical and chemical properties of soils affect metal mobilization/immobilization processes, among which grain size is one of the most significant factors [[55], [56], [57]]. Previous studies indicated that metals of natural origin were more closely associated with soil properties, such as soil texture [58,59]. Cadmium, Fe, Pb, and Zn did not correlate with soil texture, indicating their anthropogenic origin. DTPA-extractable content of Co, Fe, Mn, and Ni displayed a significant negative correlation with soil pH (Table 3), even though the studied soils are fairly balanced in terms of this parameter (Table 1). Simultaneously, bioavailable Co, Fe, and Mn forms were significantly and positively correlated with soil OM. In weakly acidic to alkaline soil conditions (pH 6–8), PTEs mobility is increased in soils with higher OM content due to the formation of soluble complexes [60].

The SOM analysis of PTEs' distribution characteristics among superclasses (Fig. 4) shows that superclass 1, comprising ten soil samples from the northern and western parts of the investigated area (Fig. 3, a), has the highest mean concentrations of Co, Cu, and Mn among classes. According to Barrett's classification [46], the concentrations of Cu and Mn in this superclass are very high. Superclass 2, which contains ten samples collected in the closest vicinity of the plant, shows very high mean concentrations of Mn and high concentrations of Cu and Fe. Superclass 3 contains ten samples, mainly from the northeast area, with high Cu and Mn concentrations. Superclass 4 comprises eight samples from the northeast of the investigated area with the highest mean concentrations of Cd, Ni, Pb, and Zn among the classes, confirming the common source of these elements. The detailed visualization of variables and observations derived by SOMs revealed the homology in Cd, Pb, and Zn distribution, thus confirming their common origin. The advantage of SOM over other multivariate methods in revealing the correlation and classification of different variables and observations, especially for high-dimensional and complex data sets, is proven in previous studies [36]. This method can provide more reliable results in complex and linear problems due to their robustness to noise data and extreme values in complex high-dimensional datasets. The SOM uses unsupervised learning, hence does not require previous information on the dataset, which is also an advantage of this method compared to other clustering methods.

The multivariate methods, PCA and PMF, indicated the sources of PTEs in soils of the investigated area. The PCA-extracted factor 1 showed high loadings of OM, pH, Co, Fe, and Mn (Table 4). Factor 2 comprises the high loadings of Cd, Ni, Pb, and Zn, pointed out their common origin probably from the steel production process. Factor 3, which shows high factor loadings of clay and Cu, could be characterized as geogenic. According to the grouping obtained by PMF (Fig. 5), factors 1 and 4 could be recognized as mainly anthropogenic factors, and factors 2 and 3 as natural/geogenic and mixed. Compared to PCA, PMF generates a more reliable representation of elements in source profiles by including a quantitative contribution of each element. Unlike PCA, which relies on the variables' correlations, raw data in PMF make it more sensitive to the more minor changes in the data. Conversely, the PMF method has the advantage of positivity constraints, providing the number of sources and source uncertainties over PCA [61]. The single-method source analysis could derive the imprecise evaluation of contamination sources, leading to the disputation of the reliability of model results. Therefore, it is advantageous to apply multiple receptor models to achieve reliable source identification and apportionment [62,63]. All multivariate analysis methods identified the common origin of Cd, Zn, and Pb, likely from a steel production plant. This finding follows similar research worldwide which identified these elements as reliable indicators for source apportionment in steel production areas [3,51,54,64]. Iron coal storage and unprotected transportation may contribute to soil pollution surrounding steel production facilities [65]. Vehicle emissions and abrasion of road asphalt because of vehicular movement could also contribute to Pb [66,67] and Cd [68] pollution. The increased pseudototal concentrations of trace elements of Mn, Ni, and Fe have been reported for soils surrounding steelmaking facilities in Australia [1], with higher concentrations reported for soils near blast furnace integrated operations compared to those near electric arc once. Although within the acceptable limits of local regulations, the pseudototal concentrations of Fe, Cu, and Ni in some soils surrounding the Australian steelmaking facilities exceeded the threshold values for acceptable soil quality, according to US EPA [69] and Canadian soil quality [70] guidelines. Font et al. (2022) identified stainless-steel processes as one of the main contributors to Ni concentrations in an industrial area in the United Kingdom [71]. Although most literature suggests that Fe is of geogenic origin, steel production facilities were also identified as contributors to Fe concentrations mainly due to the blast furnaces' operation [72,73]. An extensive study on topsoil quality assessment near a closed steel smelter at the Capital Iron and Steel Factory, Beijing in China, confirmed that higher concentrations of Cu in soils surrounding the steelmaking site are mainly associated with steel smelting activities [4].

Data on bioavailable PTEs' concentrations and distribution around the steelmaking facility provide a base for future soil monitoring activities enabling the detection of increasing pollution risk due to the ineffectiveness of applied protective measures, the change in production processes, and other factors. The spatial distribution of the PTEs obtained by geostatistics and correlation analysis and source apportionment derived by two multivariate analysis methods yielded consistent results. However, including the samples in the plant's immediate vicinity will enable the assessment of factors contributing to the area's pollution other than stack emissions, such as ore stocking, ore and slag transportation ways and routes, and deposition of slag material. Furthermore, the extensive sampling campaign, which will include the other pollution sources in the Smederevo industrial area, would improve the assessment results, enabling the delineation of different industrial sources contributing to overall environmental pollution. The differences in toxicity, bioavailability, migration mechanisms, and persistence among different forms of PTEs should be included in assessing PTEs' impacts on human health and the environment. Such a comprehensive approach will be helpful for assessing the environmental risk of contaminated sites and as a basis for soil remediation.

## Author contribution statement

Snežana Dragović, Ivana Smičiklas: Conceived and designed the experiments; Performed the experiments; Contributed reagents, materials, analysis tools or data; Analyzed and interpreted the data; Wrote the paper.

Ranko Dragović, Antonije Onjia: Conceived and designed the experiments; Analyzed and interpreted the data; Contributed reagents, materials, analysis tools or data; Wrote the paper.

Mihajlo Jović, Aleksandar Čupić, Boško Gajić: Performed the experiments; Contributed reagents, materials, analysis tools or data; Analyzed and interpreted the data; Wrote the paper.

## Data availability statement

Data included in article/supplementary material/referenced in article.

## Declaration of competing interest

The authors declare that they have no known competing financial interests or personal relationships that could have appeared to influence the work reported in this paper.
