# Supplementary material for: Spatial distribution and source apportionment of DTPA-extractable metals in soils surrounding the largest Serbian steel production plant
Source: Heliyon. 2023 May 15;9(5):e16307. doi: 10.1016/j.heliyon.2023.e16307 (PMC10209400; doi:10.1016/j.heliyon.2023.e16307)
Supplement: Multimedia component 1 [file mmc1.docx]

**Supplementary Material**

Spatial distribution and source apportionment of DTPA-extractable metals in soils surrounding the largest Serbian steel production plant

Snežana Dragović^a^, Ivana Smičiklas^a^, Mihajlo Jović^a^, Aleksandar Čupić^a^, Ranko Dragović^b^, Boško Gajić^c^, Antonije Onjia^d^

a"VINČA" Institute of Nuclear Sciences – National Institute of the Republic of Serbia, University of Belgrade, Mike Petrovića Alasa 12-14, 11351 Belgrade, Serbia

bUniversity of Niš, Faculty of Sciences and Mathematics, Department of Geography, Višegradska 33, 18000 Niš, Serbia

cFaculty of Agriculture, University of Belgrade, Nemanjina 6, 11080 Belgrade, Serbia

dFaculty of Technology and Metallurgy, University of Belgrade, 11120 Belgrade, Serbia


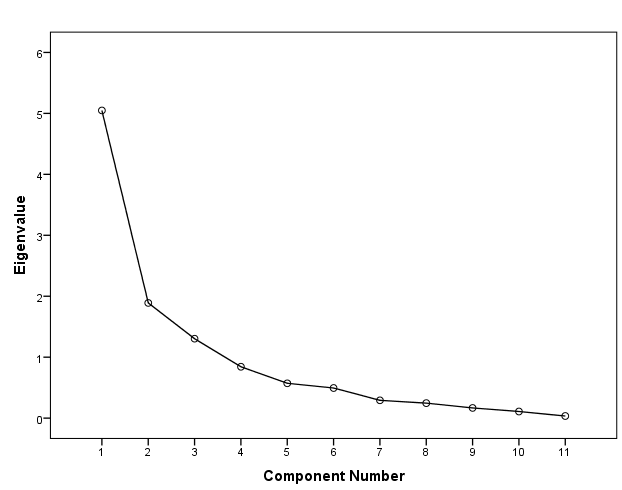


Figure S1. Eigen analysis of the correlation matrix (scree plot), related to Table 4

Table S1. Geographic coordinates of sampling sites, related to Figure 1

| Sample | Latitude | Longitude |
| --- | --- | --- |
| 1 | 44.5918 | 20.9581 |
| 2 | 44.5898 | 20.9497 |
| 3 | 44.5859 | 20.9488 |
| 4 | 44.5856 | 20.9102 |
| 5 | 44.5863 | 20.9144 |
| 6 | 44.5897 | 20.9793 |
| 7 | 44.6065 | 21.0081 |
| 8 | 44.6065 | 20.9787 |
| 9 | 44.6120 | 20.9760 |
| 10 | 44.6250 | 20.9795 |
| 11 | 44.6134 | 20.9605 |
| 12 | 44.5959 | 20.9664 |
| 13 | 44.5931 | 20.9609 |
| 14 | 44.5947 | 20.9583 |
| 15 | 44.5971 | 20.9573 |
| 16 | 44.5988 | 20.9561 |
| 17 | 44.5977 | 20.9534 |
| 18 | 44.6306 | 20.9724 |
| 19 | 44.6366 | 20.9709 |
| 20 | 44.6348 | 20.9818 |
| 21 | 44.6098 | 20.9459 |
| 22 | 44.6117 | 20.9481 |
| 23 | 44.6124 | 20.9549 |
| 24 | 44.5954 | 20.9858 |
| 25 | 44.6010 | 20.9918 |
| 26 | 44.6019 | 20.9845 |
| 27 | 44.6060 | 20.9841 |
| 28 | 44.6144 | 20.9699 |
| 29 | 44.6254 | 20.9856 |
| 30 | 44.6274 | 20.9881 |
| 31 | 44.6253 | 20.9909 |
| 32 | 44.6225 | 20.9951 |
| 33 | 44.6198 | 20.9998 |
| 34 | 44.6200 | 20.9790 |
| 35 | 44.6179 | 20.9778 |
| 36 | 44.6158 | 20.9769 |
| 37 | 44.6218 | 20.9606 |
| 38 | 44.6075 | 20.9601 |

Table S2. Basic soil characteristics in the steel production facility’s surroundings, related to Table 1

| Sample | Sand | Silt | Clay | OM | pH |
| --- | --- | --- | --- | --- | --- |
|  | % | | | |  |
| 1 | 12.7 | 64.5 | 22.8 | 2.39 | 7.52 |
| 2 | 42.4 | 36.6 | 21.0 | 1.22 | 7.84 |
| 3 | 23.4 | 52.4 | 24.2 | 2.39 | 6.48 |
| 4 | 5.5 | 64.5 | 30.0 | 1.95 | 7.19 |
| 5 | 4.1 | 63.4 | 32.5 | 1.31 | 5.94 |
| 6 | 36.3 | 46.7 | 17.0 | 1.77 | 7.66 |
| 7 | 46.4 | 26.2 | 27.4 | 1.30 | 6.79 |
| 8 | 36.3 | 42.9 | 20.8 | 0.69 | 8.00 |
| 9 | 16.0 | 55.3 | 28.7 | 1.40 | 7.52 |
| 10 | 34.2 | 39.4 | 26.4 | 1.86 | 7.42 |
| 11 | 9.1 | 60.7 | 30.2 | 0.76 | 7.98 |
| 12 | 12.8 | 60 | 27.2 | 2.06 | 7.53 |
| 13 | 23.0 | 57.6 | 19.4 | 2.50 | 7.61 |
| 14 | 5.40 | 63.2 | 31.4 | 2.38 | 7.13 |
| 15 | 7.40 | 63.7 | 28.9 | 2.72 | 7.48 |
| 16 | 31.6 | 46.8 | 21.6 | 1.17 | 7.63 |
| 17 | 4.3 | 68.4 | 27.3 | 3.19 | 6.73 |
| 18 | 9.7 | 62.9 | 27.4 | 1.90 | 7.48 |
| 19 | 34.2 | 47.8 | 18.0 | 1.96 | 7.84 |
| 20 | 44.0 | 36.3 | 19.7 | 1.96 | 7.12 |
| 21 | 4.8 | 71.7 | 23.5 | 3.36 | 6.07 |
| 22 | 7.1 | 69.9 | 23.0 | 2.06 | 7.77 |
| 23 | 5.3 | 74.6 | 20.1 | 3.13 | 7.00 |
| 24 | 8.9 | 69.9 | 21.2 | 2.55 | 7.87 |
| 25 | 19.8 | 58.3 | 21.9 | 1.30 | 7.84 |
| 26 | 17.6 | 65.6 | 16.8 | 1.66 | 7.82 |
| 27 | 12.2 | 68.6 | 19.2 | 2.17 | 7.41 |
| 28 | 16.7 | 69.3 | 14.0 | 1.05 | 8.00 |
| 29 | 32.3 | 39.4 | 28.3 | 1.42 | 7.74 |
| 30 | 25.6 | 45.3 | 29.1 | 1.64 | 7.80 |
| 31 | 35.2 | 39.3 | 25.5 | 2.65 | 6.98 |
| 32 | 31.5 | 40.8 | 27.7 | 2.08 | 7.52 |
| 33 | 26.1 | 47.1 | 26.8 | 4.44 | 6.94 |
| 34 | 48.3 | 28.6 | 23.1 | 2.06 | 7.07 |
| 35 | 20.8 | 56.4 | 22.8 | 0.92 | 8.04 |
| 36 | 24.9 | 56.8 | 18.3 | 2.21 | 7.73 |
| 37 | 7.1 | 66.1 | 26.8 | 1.72 | 7.63 |
| 38 | 6.7 | 72.7 | 20.6 | 2.63 | 7.10 |

Table S3. DTPA-extractable PTE concentrations in soil surrounding steel production facility, related to Table 1

| Sample | Cd | Co | Cu | Fe | Mn | Ni | Pb | Zn |
| --- | --- | --- | --- | --- | --- | --- | --- | --- |
|  | mg kg^-1^ | | | | | | | |
| 1 | 0.051±0.002 | 0.107±0.004 | 1.55±0.06 | 9.99±0.58 | 34.0±1.8 | 1.11±0.04 | 5.00±0.21 | 2.08±0.07 |
| 2 | 0.014±0.001 | 0.006±0.000 | 1.67±0.03 | 12.7±0.4 | 17.2±0.6 | 1.17±0.03 | 2.91±0.15 | 3.05±0.16 |
| 3 | 0.062±0.002 | 0.147±0.007 | 2.32±0.09 | 53.6±1.9 | 54.0±2.7 | 3.73±0.16 | 2.31±0.09 | 2.29±0.10 |
| 4 | 0.067±0.003 | 0.086±0.006 | 6.84±0.31 | 31.2±1.3 | 40.8±1.5 | 1.76±0.04 | 1.75±0.04 | 2.72±0.13 |
| 5 | 0.093±0.005 | 0.188±0.010 | 8.12±0.32 | 89.2±5.1 | 88.7±5.2 | 2.81±0.11 | 1.95±0.08 | 3.56±0.15 |
| 6 | 0.063±0.002 | 0.109±0.003 | 2.04±0.17 | 26.4±1.0 | 34.2±1.1 | 1.67±0.05 | 1.79±0.07 | 7.04±0.28 |
| 7 | 0.037±0.002 | 0.053±0.002 | 4.23±0.13 | 97.7±3.8 | 37.9±1.9 | 15.2±0.66 | 2.68±0.12 | 2.36±0.08 |
| 8 | 0.009±0.000 | 0.040±0.001 | 0.602±0.020 | 8.29±0.38 | 15.8±0.6 | 0.583±0.012 | 0.864±0.031 | 1.29±0.05 |
| 9 | 0.082±0.003 | 0.018±0.001 | 4.66±0.19 | 24.6±0.9 | 29.8±1.2 | 3.98±0.14 | 1.80±0.07 | 5.01±0.21 |
| 10 | 0.058±0.003 | 0.104±0.003 | 1.90±0.15 | 19.1±0.8 | 28.2±1.0 | 2.03±0.09 | 1.82±0.09 | 9.27±0.33 |
| 11 | 0.084±0.002 | 0.030±0.001 | 3.41±0.14 | 11.9±0.4 | 14.2±0.6 | 0.572±0.03 | 2.21±0.10 | 5.90±0.28 |
| 12 | 0.056±0.005 | 0.078±0.003 | 2.21±0.10 | 21.7±1.3 | 27.7±0.9 | 1.56±0.09 | 1.42±0.06 | 1.50±0.08 |
| 13 | 0.091±0.003 | 0.092±0.005 | 1.72±0.06 | 21.4±0.9 | 23.9±1.2 | 1.43±0.06 | 2.13±0.07 | 3.47±0.14 |
| 14 | 0.053±0.001 | 0.169±0.006 | 1.32±0.04 | 14.2±0.8 | 47.6±2.1 | 1.35±0.04 | 1.27±0.05 | 0.882±0.023 |
| 15 | 0.057±0.002 | 0.141±0.005 | 28.4±2.1 | 15.4±0.6 | 42.9±2.3 | 1.40±0.04 | 1.65±0.09 | 1.40±0.07 |
| 16 | 0.018±0.001 | 0.052±0.001 | 1.01±0.03 | 8.66±0.51 | 21.7±0.8 | 0.573±0.009 | 0.814±0.030 | 0.617±0.022 |
| 17 | 0.089±0.004 | 0.209±0.012 | 2.54±0.11 | 40.6±1.7 | 65.9±2.8 | 2.41±0.12 | 3.64±0.11 | 2.25±0.11 |
| 18 | 0.055±0.002 | 0.178±0.010 | 10.7±0.63 | 14.4±0.6 | 53.6±1.8 | 3.89±0.21 | 1.56±0.06 | 1.62±0.06 |
| 19 | 0.028±0.002 | 0.047±0.003 | 1.78±0.06 | 8.46±0.32 | 22.8±0.8 | 0.467±0.011 | 1.99±0.12 | 1.46±0.06 |
| 20 | 0.046±0.003 | 0.101±0.004 | 6.09±0.35 | 44.9±2.0 | 33.1±1.3 | 4.19±0.19 | 2.28±0.08 | 2.03±0.09 |
| 21 | 0.091±0.003 | 0.474±0.017 | 1.78±0.07 | 69.5±2.8 | 89.1±3.7 | 2.75±0.08 | 1.69±0.06 | 2.90±0.13 |
| 22 | 0.052±0.002 | 0.131±0.007 | 3.73±0.14 | 10.6±0.7 | 28.0±1.1 | 1.17±0.03 | 1.68±0.05 | 1.32±0.05 |
| 23 | 0.107±0.004 | 0.213±0.014 | 4.41±0.19 | 32.5±1.1 | 57.2±2.0 | 2.28±0.10 | 2.05±0.08 | 3.48±0.17 |
| 24 | 0.071±0.002 | 0.052±0.002 | 1.65±0.06 | 16.1±0.7 | 24.4±1.2 | 1.43±0.06 | 2.12±0.10 | 2.34±0.10 |
| 25 | 0.034±0.001 | 0.089±0.003 | 2.30±0.14 | 14.7±0.8 | 24.8±0.8 | 1.66±0.06 | 1.51±0.06 | 1.78±0.06 |
| 26 | 0.071±0.004 | 0.103±0.002 | 3.99±0.12 | 26.9±0.9 | 34.1±1.7 | 1.90±0.08 | 2.27±0.14 | 5.82±0.26 |
| 27 | 0.102±0.005 | 0.269±0.009 | 2.26±0.11 | 15.6±0.5 | 59.7±2.6 | 3.05±0.11 | 2.50±0.08 | 3.14±0.16 |
| 28 | 0.084±0.004 | 0.067±0.002 | 1.80±0.05 | 10.0±0.3 | 24.2±1.5 | 1.22±0.04 | 2.50±0.06 | 1.54±0.06 |
| 29 | 0.064±0.002 | 0.038±0.001 | 2.64±0.14 | 15.7±0.8 | 19.6±0.7 | 2.52±0.10 | 4.75±0.21 | 1.96±0.08 |
| 30 | 0.126±0.007 | 0.079±0.005 | 3.83±0.19 | 19.9±1.0 | 28.4±1.8 | 3.30±0.13 | 6.89±0.31 | 5.41±0.29 |
| 31 | 0.091±0.003 | 0.196±0.010 | 2.88±0.14 | 45.5±1.8 | 50.7±2.8 | 9.08±0.37 | 4.48±0.22 | 2.77±0.12 |
| 32 | 0.386±0.011 | 0.059±0.002 | 4.81±0.21 | 42.9±2.4 | 32.9±1.2 | 4.41±0.18 | 17.7±0.7 | 14.0±0.6 |
| 33 | 0.140±0.008 | 0.147±0.006 | 3.87±0.16 | 90.0±3.8 | 40.7±1.7 | 9.20±0.41 | 6.11±0.28 | 6.00±0.34 |
| 34 | 0.042±0.001 | 0.109±0.004 | 2.47±0.08 | 32.6±1.2 | 30.9±1.1 | 8.27±0.33 | 2.84±0.07 | 3.33±0.17 |
| 35 | 0.026±0.002 | 0.028±0.001 | 2.03±0.06 | 11.7±0.3 | 16.0±0.6 | 0.590±0.014 | 1.56±0.07 | 0.81±0.02 |
| 36 | 0.060±0.002 | 0.133±0.004 | 1.13±0.04 | 8.97±0.52 | 37.6±1.2 | 1.26±0.07 | 2.41±0.13 | 3.07±0.12 |
| 37 | 0.041±0.001 | 0.100±0.004 | 1.69±0.09 | 17.1±0.9 | 27.6±1.3 | 1.11±0.03 | 1.01±0.04 | 1.57±0.09 |
| 38 | 0.086±0.005 | 0.183±0.011 | 2.15±0.10 | 42.2±2.2 | 51.9±2.0 | 2.57±0.11 | 2.36±0.12 | 2.61±0.11 |
